# Generation and Detection of Levuglandins and Isolevuglandins *In Vitro* and *In Vivo*

**DOI:** 10.3390/molecules16075333

**Published:** 2011-06-24

**Authors:** Ming Zhang, Wei Li, Tao Li

**Affiliations:** 1 Reproductive Medicine Center, Zhongnan Hospital of Wuhan University, Wuhan 430071, China; E-Mail: mjiinag@yahoo.com.cn (M.Z.); 2 Office of the Texas State Chemist, Texas A&M University, College Station, TX 77845, USA; E-Mail: wei@otsc.tamu.edu (W.L.); 3 Department of Ophthalmology, Tongji Hospital, Tongji Medical College, Huazhong University of Science and Technology, Wuhan 430030, China

**Keywords:** levuglandin, polyunsaturated fatty acids, oxidative injury

## Abstract

Levuglandins (LGs) and isolevuglandins (isoLGs), formed by rearrangement of endoperoxide intermediates generated through the cyclooxygenase and free radical induced oxidation of polyunsaturated fatty acids (PUFAs), are extraordinarily reactive, forming covalent adducts incorporating protein lysyl ε-amino groups. Because they accumulate, these adducts provide a dosimeter of oxidative injury. This review provides an updated and comprehensive overview of the generation of LG/isoLG *in vitro and in vivo* and the detection methods for the adducts of LG/isoLG and biological molecules *in vivo*.

## 1. Introduction

Lipids are essential cell membrane components. They incorporate abundant polyunsaturated fatty acids (PUFAs) that are particularly susceptible to oxidative damage. In view of the ready oxidizability of PUFAs and the aerobic environment we live in, it is understandable that lipid oxidation plays a key role in human health. Increasing substantial evidence suggests that lipid oxidation is involved in the development of many chronic diseases. This has stimulated worldwide efforts to elucidate the mechanisms and pathological consequences of lipid oxidation [1,2,3].

Levuglandins (LGs) and isolevuglandins (isoLGs)—also referred to as the “isoketals” [4]—are γ-ketoaldehydes that are formed by rearrangement of endoperoxide intermediates generated through the cyclooxygenase and free radical-induced oxidation of arachidonates. The γ-ketoaldehyde functionality of the LGs and isoLGs makes them extraordinarily reactive towards primary amino groups in biomolecules. LGs and isoLGs react with the ε-amino groups of lysyl residues in proteins to produce covalent adducts with greater avidity than most other lipid oxidation products, e.g., 4-hydroxynonenal (4-HNE) or malondialdehyde (MDA) [4]. This feature makes covalent LG/isoLG adducts attractive as biomarkers to evaluate oxidative injury in the tissues. LGs/isoLGs initially react with primary amino groups to form in seconds Schiff base adducts which are transformed to pyrrole adducts in minutes. However, these highly alkylated pyrroles are chemically sensitive compounds in the presence of oxygen [5] and are further oxidized in a few hours to stable end products, lactams and hydroxylactams (HLs) [6]. First detected *in vivo* by immunoassays, quantitative analysis of LG/isoLG protein adducts was also achieved by LC-MS after proteolysis to LG/isoLG-lysine derivatives that incorporate the lysine ε-amino group into LG/isoLG-derived lactams. Salomon *et al*. reported that isoLGE_2_-protein [7], iso[4]LGE_2_-protein [8] and iso[7]LGD_2_-protein [9] adducts are generated upon oxidation of LDL *in vitro*. Formation of isoLG protein adducts *in vivo* has also been confirmed by a variety of immunological and mass spectrometric methods [10,11]. The levels of isoLG-protein adducts are elevated in diseases associated with oxidative injury [11].

This review will describe levuglandins and isolevuglandins with emphasis on their generation, protein modification and identification of the novel and stable compounds produced during the oxidation of PUFAs which could serve as unique indicators for PUFA-associated oxidative injuries, and provide valuable insights into the pathophysiology of diseases related to oxidative stress.

## 2. Generation of Levuglandins and Isolevuglandins

Arachidonic acid, a linear twenty carbon methylene interrupted polyunsaturated fatty acid (C20:4ω6), is oxidatively transformed by two different enzymatic metabolic pathways catalyzed by cyclooxygenase and lipoxygenase, resulting in a large array of biologically active oxidized 20 carbon fatty acids, collectively called eicosanoids. Besides these enzymatic pathways, growing evidence has also suggested the existence of a free radical-induced metabolic pathway which further greatly expands the family of eicosanoids [9,12,13,14,15,16].

### 2.1. Enzymatic Pathways

Cyclooxygenase can catalyze a unique transformation, in which two molecules of oxygen are chemoselectively and stereospecifically added to a molecule of AA to produce prostaglandin endoperoxide G_2_ (PGG_2_). This PGG_2_ is a highly reactive molecule which undergoes chemoselective reduction catalyzed by hydroperoxidase, to produce the unstable prostaglandin endoperoxide H_2_ (PGH_2_, t_1/2_ = 5 min at 37 °C in aqueous solution) [17]. PGH_2_ is a pivotal intermediate in regulating a wide variety of cellular activities and undergoes enzymatic and non enzymatic (solvent-induced) rearrangements to provide an array of physiologically active molecules. Lipoxygenases, on the other hand, can catalyze the conversion of polyunsaturated fatty acids (PUFAs) into the corresponding fatty acid hydroperoxides by insertion of a molecule of oxygen, for example, soybean lipoxygenase, can generate 13-hydroperoxyoctadecadienoate (13-HPODE) from linoleic acid (LA) and 15-hydroperoxy-eicosatetraenoate (15-HPETE) from AA.

The major products of the enzymatic rearrangement of PGH_2_ are prostaglandin E_2_ (PGE_2_), prostaglandin D_2_ (PGD_2_), prostaglandin and thromboxane A_2_ (TXA_2_). The solvent-induced nonenzymatic conversion also results in formation I_2_ (PGI_2_) from PGE_2_ and PGD_2_. Reduction of PGH_2_ results in prostaglandin F_2α_ (PGF_2α_) [18,19]. Enzyme-induced skeletal rearrangement of the strained bicyclic ring system of PGH_2_ results in PGI_2_ and TXA_2_. 12-Hydroxyheptadeca-5(*Z*),8(*E*),10(*E*)-trienoic acid (HHT) and malondialdehyde (MDA) are produced as a result of fragmentation of PGH_2_ [20,21]. These molecules exert various physiological effects that are often complimentary to each other [22,23,24,25,26,27].

Salomon [28,29] discovered that a novel alternative rearrangement of PGH_2_ also occurs that produces two γ-ketoaldehydes, which were named levuglandin E_2_ (LGE_2_) and levuglandin D_2_ (LGD_2_) because they are derivatives of levulinaldehyde with prostanoid side chains [7]. Detailed mechanistic studies revealed that levulinaldehyde was generated as a result of a 1,2-hydride shift during the cleavage of three bonds in a concerted fashion with a polarized transition state during the rearrangements of endoperoxide in aqueous solution [30,31].

**Scheme 1:**
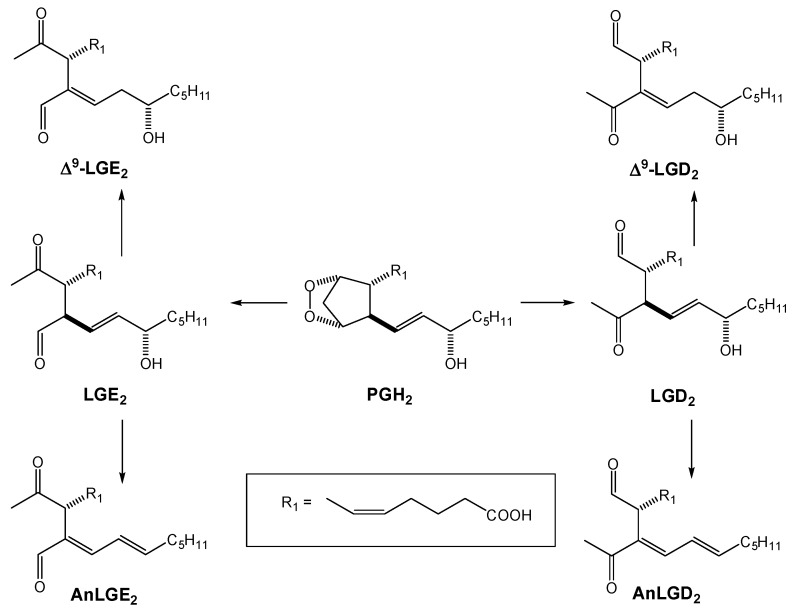
Decomposition of PGH_2_: Generation of levuglandins, anhydrolevuglandins and Δ^9^-levuglandins.

Levuglandins are sensitive vinylogous β-hydroxy carbonyl compounds which readily undergo dehydration leading to their anhydro analogs AnLGE_2_ and AnLGD_2_ as shown in Scheme 1.

The propensity of LGD_2_ to undergo dehydration has precluded its isolation as well as total synthesis [29]. The C10-C11 double bond also migrates to form the more stable conjugated isomers, Δ^9^-LGE_2_ and Δ^9^-LGD_2_ [30]. Convergent asymmetric total syntheses of LGE_2_ and AnLGD_2_ were developed that not only confirmed their structures but also provided adequate amounts of these complex AA metabolites for thorough chemical and biological studies [32,33].

### 2.2. Free Radical Pathways

Generation of stereo and structural isomers of PGs was firstly recognized in a study to quantify the biologically relevant PGs generated enzymatically through the action of COX during AA metabolism [34]. However, the biological significance of this pathway, now called the isoprostane pathway, was ignored for two decades.

In the early 90s, researchers reported that an *in vivo* non enzymatic generation of PGH_2_ isomers occurs in humans resulting in the generation of large amounts of PG isomers upon AA metabolism [12]. The *in vitro* formation of significant amounts of 8-*epi*-PGF_2α_ isomers as well as other isomers of PGs, named isoprostanoids, in aged human plasma, first prompted the rediscovery of this nonenzymatic pathway (isoprostane pathway). Further studies showed the *formation* of these isoprostonoids *in vivo* [13]. The generation of large amounts of 8-*epi*-PGF_2α_ and other isoprostanoids in the plasma of mice treated with free radical initiators like CCl_4_ and diquat, further supported a free radical peroxidative mechanism [14]. The fact that the generation of these isoprostanoids could not be suppressed *in vivo*, even after application of cyclooxygenase inhibitors, strongly supports the alternative, free radical pathway [35]. The same researchers also reported that isoprostanes that are PGD_2_- and PGE_2_-like compounds (D_2_/E_2_-IsoPs) were found to be generated *in vivo* as phospholipid esters through non-enzymatic rearrangements of stereo and structural isomers of PGH_2_ derivatives (isoprostane endoperoxides) that are produced through free radical-induced cyclooxgenation of arachidonyl phosphatidylcholine (AA-PC) [36].

**Scheme 2:**
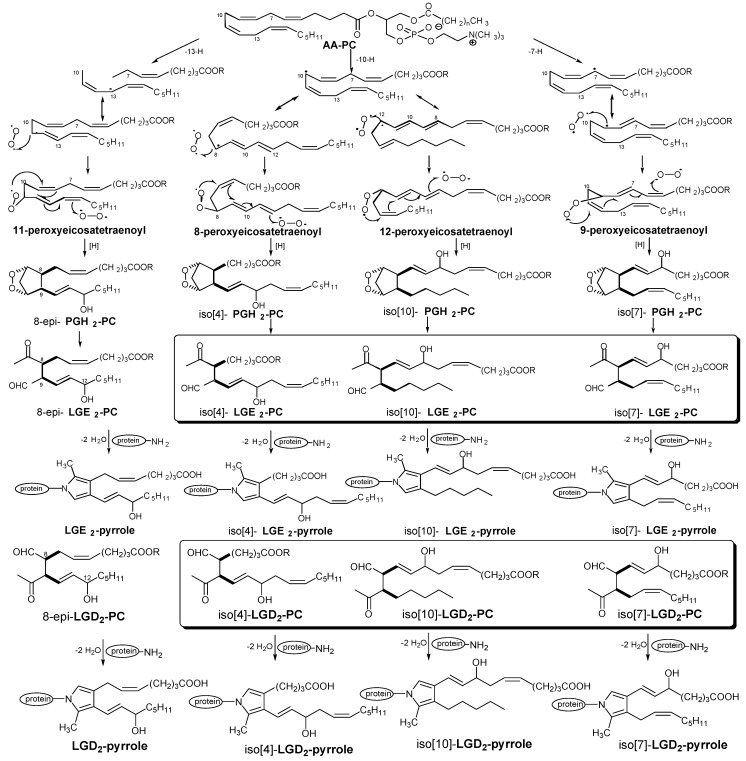
Generation of isolevuglandins and isolevuglandin-protein adducts during free radical oxidation of AA and AA-esters.

The formation of LGs during nonenzymatic rearrangement of PGH_2_ to PGs was discovered by Salomon *et al.* [37]. Based on these observations, they proposed that nonenzymatic *in vivo* generation of LG isomers from endoperoxides could occur during free radical-induced oxidation [7] (Scheme 2). The rearrangement of the 2-lysophosphatidylcholine (PC) ester 8-*epi*-PGH_2_-PC delivers 8-*epi*-LGE_2_-PC. The 8-*epi*-LGE_2_-PC isomer produced in this way is expected to differ in configuration at carbon C8 from that produced by the rearrangement of PGH_2_ generated by the cyclooxygenase pathway. It is also expected to be racemic. However, an immunochemical assay (*vide infra*) for protein-bound LG-derived pyrrole would not differentiate between cyclooxygenase-derived or nonenzymatically produced LG isomers as the stereogenic centers at both C8 and C9 in 8-*epi*-LGE_2_-PC are destroyed during LGE_2_-pyrrole formation.

Furthermore, because hydrogen atom abstraction readily occurs nonregioselectively at any doubly allylic methylene, the free radical pathway can not only produce a stereoisomer mixture of levulinaldehyde derivatives with PG side chains, *i.e.*, isoLGs, but also levulinaldehyde derivatives with non-prostanoid side chains, *i.e.*, iso[n]LGs. Non-regioselective hydrogen atom abstraction from the 7, 10, and 13-positions of an arachidonyl ester produces three regioisomeric pentadienyl radicals (Scheme 2). These then react with molecular oxygen to afford four regioisomeric peroxyeicosa-tetraenoyl radicals that undergo peroxyradical cyclization [38] to deliver four structurally isomeric endoperoxides. Each endoperoxide rearranges to form two LGs or iso[n]LGs, designated as E series if the acetyl substituent is nearer than the formyl substituent to the carboxyl group or as D series if the formyl is nearer than the acetyl to the carboxyl. For iso[n]LGs, the bracketed integers indicate the length of the carboxylic side chain [7]. For example, hydrogen atom abstraction from the 10-position of AA-PC followed by cyclization of an intermediate 8- radical could lead to *iso*[4]PGH_2_-PC and then iso[4]LGE_2_-PC, where the number in brackets signifies the length of the carboxylic side chain appended to a common 2,3-dioxabicyclo[2.2.1] heptane or levulinaldehyde core.

The terms “isoketal or IsoK” were used [39] as alternatives to the original isoLG nomenclature [7] “to distinguish them from levuglandins formed by rearrangement of the cyclooxygenase endoperoxide intermediate, PGH_2_”. However, such a distinction is erroneous because the exact same levuglandin molecules, LGE_2_ and LGD_2_, are generated by both the cyclooxygenase and isoprostane pathways. The difference between the pathways is that stereo and structural isomers are cogenerated with LGE_2_ and LGD_2_ in free radical-induced autoxidation of arachidonates. On the other hand, it is important to emphasize the difference between stereoisomers of levuglandins and structural isomers of levuglandins. In the nomenclature, this distinction is reflected in the dichotomy of isoLGs and iso[*n*]LGs. Thus, for example, LGE_2_, one of the stereoisomers designated collectively as isoLGE_2_, is a product of both the COX and isoprostane pathways whereas all stereoisomeric iso[4]LGE_2_s are only produced through the isoprostane pathway. The alternative names, 15-E2-IsoK and 12-E2-IsoK, respectively, do not convey this fundamental difference.

Studies reveal that myeloperoxidase (MPO) serves as an enzymatic catalyst for initiation of lipid peroxidation and lipoprotein oxidation *in vivo* [40]. MPO is an abundant heme protein secreted by phagocytes in response to stimulation [41]. MPO [42] and its distinct products [HOCl-damaged proteins [43] and 3-chlorotyrosine [44] are enriched in human atherosclerotic aortic intima and LDL recovered from atheroma. MPO uses H_2_O_2_ together with low molecular weight cosubstrates like chloride [45], tyrosine [46],and nitrite (NO_2_^−^) [47,48] to generate a variety of reactive oxidants and diffusible radical species [49,50]. With the MPO-H_2_O_2_-NO_2_^−^ catalytic system, Salomon group confirmed the *in vitro* generation of isoLGs, isoLGE_2_, iso[4]LGE_2_ and iso[7]LGE_2_, during the free radical-induced peroxidation of AA-PC. These facts further confirmed the feasibility of the “isolevuglandin pathway” in nonenzymatic AA metabolism.

### 2.3. Free Radical Oxidation of Docosahexaenoic Acid (DHA)

Docosahexaenoic acid (C22:6ω3) (DHA), a member of ω-3 PUFAs, is an essential requirement for the function and development of the human brain and retina [51]. While the content of DHA in human plasma LDL is low, the DHA content in brain and retina ranges from 20%–60% of total fatty acid content and is present estified in phospholipids [52]. Although the exact role of DHA is not clear, deficiency of DHA is associated with the abnormalities in brain function [53]. Similar to other PUFAs, DHA is readily oxidized *in vitro* because of the presence of six homoallylic double bonds. Considering of the structural similarity between AA and DHA, analogue isoprostanes could be generated upon the free radical-induced peroxidation of DHA. Enhanced lipid oxidation has been reported as a consequence of oxidative injuries or decrease in antioxidant capacity in human brain and retina as measured using the simple thiobarbituric acid (TBA) test [54,55]. The free radical-induced peroxidation of DHA might play an important role in the pathogenesis of neurodegenerative diseases, e.g., Alzheimer’s disease, Parkinson’s disease, *etc.* [56,57,58].

**Scheme 3:**
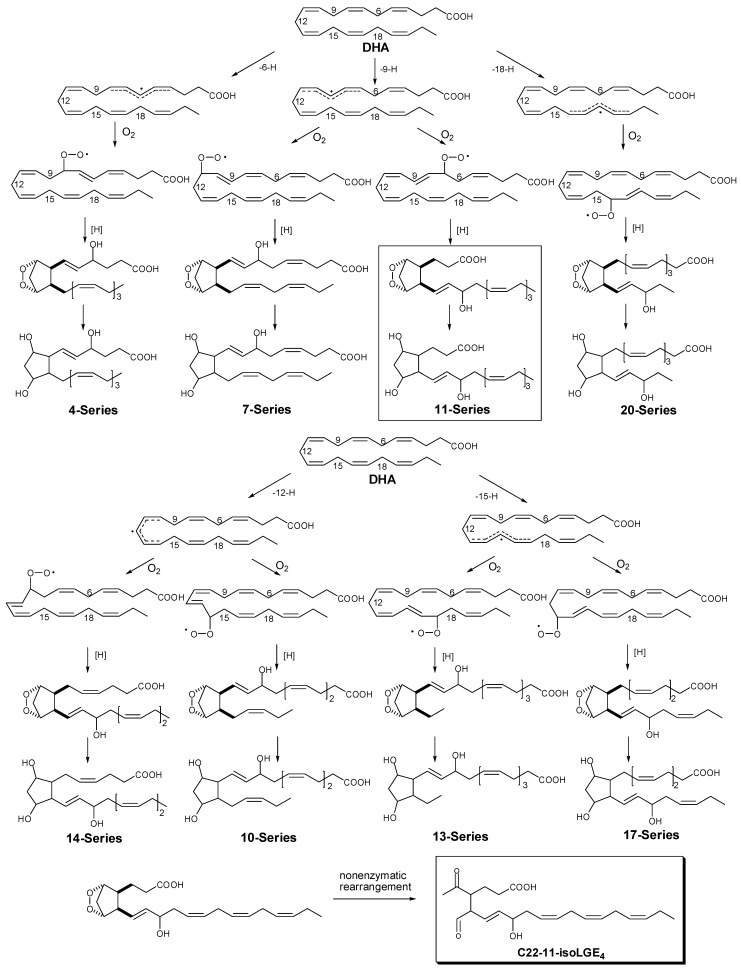
F_4_-C22-isoprostane and C22-isolevuglandin (11-isoLGE_4_) generation from nonenzymatic peroxidation of docosahexaenoic acid (DHA).

Despite the complications involved, identification of DHA peroxidation products have been carried out by several research groups [59,60].The quantification of such peroxidation products might provide a unique marker of oxidative injury in human brain and retina, and also a better understanding of their biological roles. In analogy to the free radical-induced mechanism that produces F_2_-isoprostanes from arachidonic acid, F_4_-C22-isoprostanes, 22-carbon analogues of PGF_2α_, were generated during the *in vitro* nonenzymatic peroxidation of DHA [59]. The mechanism of the formation of F_4_-C22-isoprostanes (Scheme 3) involves generation of DHA radicals at doubly allylic methylene positions, addition of molecular oxygen, followed by the formation of bicyclic endoperoxide intermediates from the resulting peroxyl radicals. The bicyclic endoperoxide intermediates are then reduced to form F_4_-C22-isoprostanes. It is known from previous studies [61] that such bicyclic endoperoxides can also readily undergo another kind of rearrangement to produce levuglandin-like products.

## 3. Detection of Biological Adducts of Isolevuglandins

### 3.1. Immunological Detection of Protein Adducts of LGs and IsoLGs

Previous studies by Salomon *et al*. had shown that the γ-ketoaldehyde functional array in LGE_2_ has an extraordinary proclivity towards rapid covalent adduction with proteins. For examples, human serum albumin binds 10 equivalents of LGE_2_ within 1 min [62]. Consistent with their earlier result, Brame confirmed the rapid consumption of LGE_2_ (t_1/2_ = 20 s) upon exposure to bovine serum albumin using mass spectrometry [63]. LGE_2_ reacts with ε-lysyl amino groups of proteins to form Schiff base adducts [64]. Pyrrole derivatives are then generated upon rapid cyclization and dehydration [65]. Further oxidation delivers lactam and hydroxylactam stable end products [63]. Pyrrole and Schiff-base derivatives of phospholipids are generated by analogous reactions with LGE_2_ stereoisomers with phosphatidyl ethanolamine [66].

Because LGs are rapidly sequestered by covalent adduction with proteins, Salomon *et al*. developed assays to detect their presence *in vivo* using antibodies that would recognize their protein adducts [67]. Existence of LGE_2_-protein adducts *in vivo* was first detected in human cerebral vasculature using polyclonal rabbit antibodies [10]. However, they noticed in these studies that they could not distinguish between a cyclooxygenase formation or a possible alternative free radical-induced autoxidative biogenesis for these LGE_2_-protein adducts *in vivo*. Thus, Salomon predicted that epitopes that would be recognized by LGE_2_-protein adduct antibodies could be generated by free radical-induced autoxidation of arachidonyl phospholipids [68]. Experimental evidence supporting this hypothesis was first provided by the demonstration that LGE_2_-protein adducts are generated upon free radical-induced autoxidation of low-density lipoprotein *in vitro* [7]. Thus, antibodies against LGE_2_-protein adducts were useful tools for detecting products of the isoprostane pathway for *in vitro* systems that do not contain cyclooxygenase activity. The discovery of isolevuglandins (also referred to as isoketals) exploited rabbit polyclonal antibodies against LGE_2_-protein adducts to detect the production of LGE_2_ through free radical-induced oxidation of LDL *in vitro* [7]. Further evidence supporting the conclusion that these immunoreactive protein modifications were derived from LGE_2_ stereoisomers (isoLGs) was then secured by mass spectroscopic analysis (vide infra) of the modified lysine excised from LDL protein through exhaustive proteolysis [63].

It is important to emphasize that distinguishing LGE_2_ production through the two entirely different biochemical pathways, COX and isoprostane, is a redoubtable challenge. The LGE_2_-protein adduct immunoreactivity detected in human cerebral vasculature was cited as an example of the localization of “isoK adducts” [69]. However, antibodies against LGE_2_-protein adducts cannot distinguish the operation of a free radical-promoted isoLG (isoK) biogenesis from a COX mediated biosynthesis through the levuglandin pathway *in vivo* unless the COX activity has been inhibited.

Structural isomers of LGE_2_, designated collectively as iso[*n*]LGE_2_s, are unique products of the free radical pathway (Scheme 2). Therefore, to provide a valuable tool for the unambiguous assessment isoLG production *in vivo*, antibodies against iso[4]LGE_2_-protein adducts [8] were raised and used to demonstrate the presence of isolevuglandins in human blood [11]. Antibodies raised against iso[7]LGD_2_-protein adducts further confirmed the operation of the isolevuglandin pathway *in vivo* [9]. The mean levels of the iso[4]LG and iso[7]LG adducts, as well as those detected with antibodies raised against LGE_2_-protein adducts [10], are elevated in plasma from individuals with atherosclerosis compared to individuals with no cardiovascular disease.

### 3.2. Mass Spectrometric Detection of Protein Adducts of LGs and IsoLGs

Mass spectrometry is increasingly being employed to identify the structures of biomolecules from *in vivo* samples, especially of proteins (proteomics), lipids (lipidomics) and metabolites (metabolomics). This is mostly owing to the discovery of soft ionization techniques, such as electrospray ionization (ESI) [70] and matrix-assisted laser desorption ionization (MALDI) [71,72] over 20 years ago. Also, the design of new more robust instruments and user-friendly software that allows multiple data processing and analysis have made mass spectrometry an accessible technique for the study of biomolecules in general. Briefly, organic mass spectrometry is an analytical technique which allows measuring the molecular weight and relative abundances of an analyte. In addition, generally by using tandem mass spectrometry, structural information of molecules can also be obtained. The most common ionization methods used in the analysis of phospholipids are electrospray ionization (ESI) and matrix-assisted laser desorption ionization (MALDI). These soft ionization methods produce almost no fragmentation, and allow the ionization of non-volatile and thermolabile samples. Usually, this is an advantage, especially in the analysis of mixtures, but only information about the molecular weight of the compounds can be obtained. However, it is possible to induce the fragmentation of the sample ions, commonly by using collision induced dissociation (CID). In this technique called tandem mass spectrometry, MS/MS, fragmentation of a selected ion by collision with an inert gas is induced and the product ions are analyzed. High sensitivity analysis, usually in the fmol range, of complex sample mixtures and the capacity of coupling with separation techniques, namely liquid chromatography (LC) are other advantages of this technique. It is beyond the scope of this review to give a comprehensive outline of the mass spectrometry fundamentals. Encompassing this emergent trend, mass spectrometry is becoming increasingly important in phospholipid oxidation research. One of the main reasons for this, other than the increasing spread of mass spectrometers, is that interpreting mass spectra of oxidized phospholipids is usually straightforward [73,74].

**Scheme 4:**
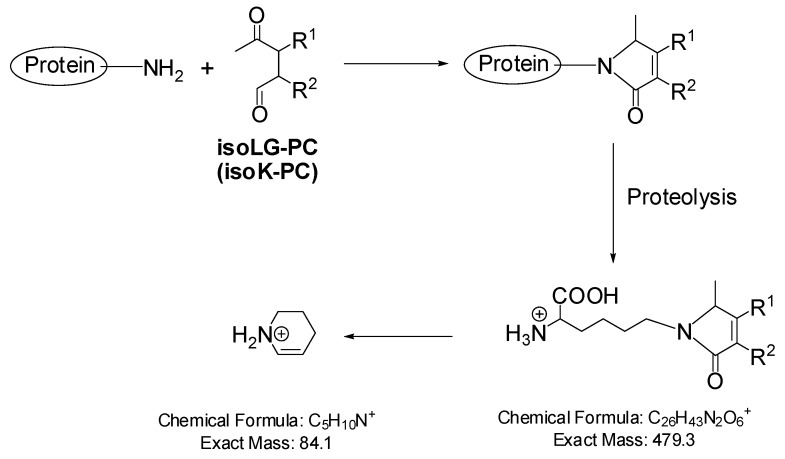
MS/MS fragmentation of isolevuglandin-derived lysyl lactams.

LC-ESI/MS/MS analysis of the lysyl lactam adduct of LGE_2_ shows a parent ion at *m*/*z* 479.3 and a daughter ion at *m*/*z* 84.1 that arises from the lysyl moiety [63]. All isoLG-derived lysyl lactams can deliver parent and daughter ions with these same exact masses (Scheme **4**). LC-ESI/MS/MS analysis of the amino acids obtained by exhaustive proteolysis of isoLG-apoB adducts, generated upon oxidation of LDL *in vitro*, indicated a complex mixture of products exhibiting the *m*/*z* 479.3–84.1 transition as expected for a family of structurally isomeric isoLG-derived lysyl lactams. As mentioned above, this experiment confirmed their earlier finding, based on immunological evidence, that isolevuglandins are generated through free radical-induced oxidation of LDL *in vitro* [7]. The LC-MS technique was also applied to the quantitative analysis of lysyl lactams obtained by exhaustive proteolysis of plasma proteins [75]. The lactam derivatives of isoLGE_2_ prepared from [^3^H_2_,^13^C_6_]-lysine were used as an internal standard. This analytical method provides a convenient measure of total LG and isoLG protein adducts, but does not distinguish COX products, *i.e.*, levuglandins, from isolevuglandins (isoketals).

The Schiff base adducts generated initially in the reaction of LGE2 with proteins can be stabilized by reduction with sodium borohydride. Subsequent exhaustive proteolysis gives reduced adducts of lysine that exhibit a dehydrated parent ion at *m/z* 467.3 [64]. MS/MS analysis of this ion produces a daughter ion with *m/z* 321.2 that probably is a protonated tetrahydrofuryl ion generated by intramolecular nucleophilic displacement of lysine.

All isoLG-derived reduced Schiff base adducts of lysine can give parent and daughter ions with these same exact masses. Recently, the LC-ESI/MS/MS technique was applied to the quantitative analysis of reduced Schiff base adducts obtained by exhaustive proteolysis of liver proteins from rats treated with CCl_4_, a model of oxidant injury to liver [75]. Schiff base adduct levels prior to base treatment were barely detectable. Apparently, more than 97% of the Schiff base adducts were incorporated in phospholipid esters. Because only free AA is a substrate for COX, these phospholipid Schiff base adducts are unique products of free radical-induced cyclooxygenation. The provenance of Schiff base adducts that are not incorporated in phospholipid esters must be determined independently because they can also be generated through the COX pathway [76].

A remarkable dichotomy between the Schiff base and lactam adducts was observed in these experiments. While the isoLG-derived Schiff base derivatives of proteins were present in the form of phospholipid esters, the lactam derivatives were present as free acids [75]. IsoLG-Schiff base adducts are intermediates that are converted into pyrroles and then lactams. Three possible alternative explanations for the preferential incorporation of Schiff base adducts into phospholipid esters are: (1) Schiff base esters are far superior substrates for esterases than lactam esters; (2) Cyclization of Schiff bases to give pyrroles is strongly disfavored by the membrane environment to which phospholipids are anchored and (3) Oxidation of pyrroles to give lactams is strongly disfavored by the membrane environment. In other words, the last two alternatives mean that: (1) Pyrrole formation from a Schiff base or (2) Pyrrole oxidation occurs much more readily for the free acid adducts than for the phospholipid adducts. It seems likely that investigation of this dichotomy will provide valuable insights into the unique environment present at the membrane/water interface.

Except for detection of protein adducts, Salomon *et al.* [77,78] report the detection *in vivo* and quantitative analysis of LG/isoLG adducts that incorporate the primary amino group of phosphatidylethanolamines (PEs) into LG/isoLG-derivsed hydroxylactams (HLs) using HPLC-MS/MS. In pilot clinical studies, the levels of isoLG-PE-HL in plasma from Age-related Macular Degeneration (AMD) patient samples are elevated compared with those in plasma from control samples. And the levels of isoLG-PE-HL are significantly elevated in murine model of alcoholic liver disease.

## 4. Conclusions

In summary, the generation of levuglandin/isolevuglandin upon free radical-induced peroxidation of polyunsaturated fatty acid plays important roles in the pathology of diseases related to oxidative stress. The discoveries of adducts of LG/isoLG and biological molecules will provide clues about the biomarkers in the field of oxidative injuries. 

## References

[1] Ames B.N. (1983). Dietary carcinogens and anticarcinogens. Oxygen radicals and degenerative diseases. Science.

[2] Montine T.J., Neely M.D., Quinn J.F., Beal M.F., Markesbery W.R., Roberts L.J., Morrow J.D. (2002). Lipid peroxidation in aging brain and Alzheimer’s disease. Free Radic. Biol. Med..

[3] Smith M.A., Perry G., Richey P.L., Sayre L.M., Anderson V.E., Beal M.F., Kowall N. (1996). Oxidative damage in Alzheimer’s. Nature.

[4] Davies S.S., Amarnath V., Montine K.S., Bernoud-Hubac N., Boutaud O., Montine T.J., Roberts L.J. (2002). Effects of reactive gamma-ketoaldehydes formed by the isoprostane pathway (isoketals) and cyclooxygenase pathway (levuglandins) on proteasome function. FASEB J..

[5] Amarnath V., Valentine W.M., Amarnath K., Eng M.A., Graham D.G. (1994). The mechanism of nucleophilic substitution of alkylpyrroles in the presence of oxygen. Chem. Res. Toxicol..

[6] Roberts L.J., Salomon R.G., Morrow J.D., Brame C.J. (1999). New developments in the isoprostane pathway: Identification of novel highly reactive gamma-ketoaldehydes (isolevuglandins) and characterization of their protein adducts. FASEB J..

[7] Salomon R.G., Subbanagounder G., Singh U., O’Neil J., Hoff H.F. (1997). Oxidation of low-density lipoproteins produces levuglandin-protein adducts. Chem. Res. Toxicol..

[8] Salomon R.G., Sha W., Brame C., Kaur K., Subbanagounder G., O’Neil J., Hoff H.F., Roberts L.J. (1999). Protein adducts of iso[4]levuglandin E2, a product of the isoprostane pathway, in oxidized low density lipoprotein. J. Biol. Chem..

[9] Poliakov E., Meer S.G., Roy S.C., Mesaros C., Salomon R.G. (2004). Iso[7]LGD2-protein adducts are abundant *in vivo* and free radical-induced oxidation of an arachidonyl phospholipid generates this D series isolevuglandin *in vitro*. Chem. Res. Toxicol..

[10] Salomon R.G., Subbanagounder G., O’Neil J., Kaur K., Smith M.A., Hoff H.F., Perry G., Monnier V.M. (1997). Levuglandin E2-protein adducts in human plasma and vasculature. Chem. Res. Toxicol..

[11] Salomon R.G., Batyreva E., Kaur K., Sprecher D.L., Schreiber M.J., Crabb J.W., Penn M.S., DiCorletoe A.M., Hazen S.L., Podrez E.A. (2000). Isolevuglandin-protein adducts in humans: Products of free radical-induced lipid oxidation through the isoprostane pathway. Biochim. Biophys. Acta.

[12] Morrow J.D., Harris T.M., Roberts L.J. (1990). Noncyclooxygenase oxidative formation of a series of novel prostaglandins: Analytical ramifications for measurement of eicosanoids. Anal. Biochem..

[13] Morrow J.D., Hill K.E., Burk R.F., Nammour T.M., Badr K.F., Roberts L.J. (1990). A series of prostaglandin F2-like compounds are produced *in vivo* in humans by a non-cyclooxygenase, free radical-catalyzed mechanism. Proc. Natl. Acad. Sci. USA.

[14] Morrow J.D., Awad J.A., Boss H.J., Blair I.A., Roberts L.J. (1992). Non-cyclooxygenase-derived prostanoids (F2-isoprostanes) are formed in situ on phospholipids. Proc. Natl. Acad. Sci. USA.

[15] Awad J.A., Morrow J.D., Takahashi K., Roberts L.J. (1993). Identification of non-cyclooxygenase-derived prostanoid (F2-isoprostane) metabolites in human urine and plasma. J. Biol. Chem..

[16] Poliakov E., Brennan M.L., Macpherson J., Zhang R., Sha W., Narine L., Salomon R.G., Hazen S.L. (2003). Isolevuglandins, a novel class of isoprostenoid derivatives, function as integrated sensors of oxidant stress and are generated by myeloperoxidase *in vivo*. FASEB J..

[17] Hamberg M., Samuelsson B. (1973). Detection and isolation of an endoperoxide intermediate in prostaglandin biosynthesis. Proc. Natl. Acad. Sci. USA.

[18] Ramwell P.W., Leovey E.M., Sintetos A.L. (1977). Regulation of the arachidonic acid cascade. Biol. Reprod..

[19] Nugteren D.H., Hazelhof E. (1973). Isolation and properties of intermediates in prostaglandin biosynthesis. Biochim. Biophys. Acta.

[20] Samuelsson B. (1983). From studies of biochemical mechanism to novel biological mediators: Prostaglandin endoperoxides, thromboxanes, and leukotrienes. Nobel Lecture, 8 December 1982. Angew. Chem. Int. Ed. Engl..

[21] Hamberg M., Svensson J., Wakabayashi T., Samuelsson B. (1974). Isolation and structure of two prostaglandin endoperoxides that cause platelet aggregation. Proc. Natl. Acad. Sci. USA.

[22] Bhagwat S.S., Hamann P.R., Still W.C., Bunting S., Fitzpatrick F.A. (1985). Synthesis and structure of the platelet aggregation factor thromboxane A2. Nature.

[23] Bunting S., Gryglewski R., Moncada S., Vane J.R. (1976). Arterial walls generate from prostaglandin endoperoxides a substance (prostaglandin X) which relaxes strips of mesenteric and coeliac ateries and inhibits platelet aggregation. Prostaglandins.

[24] Gryglewski R.J., Bunting S., Moncada S., Flower R.J., Vane J.R. (1976). Arterial walls are protected against deposition of platelet thrombi by a substance (prostaglandin X) which they make from prostaglandin endoperoxides. Prostaglandins.

[25] Hamberg M., Svensson J., Samuelsson B. (1975). Thromboxanes: A new group of biologically active compounds derived from prostaglandin endoperoxides. Proc. Natl. Acad. Sci. USA.

[26] Moncada S., Gryglewski R., Bunting S., Vane J.R. (1976). An enzyme isolated from arteries transforms prostaglandin endoperoxides to an unstable substance that inhibits platelet aggregation. Nature.

[27] Whittaker N., Bunting S., Salmon J., Moncada S., Vane J.R., Johnson R.A., Morton D.R., Kinner J.H., Gorman R.R., McGuire J.C. (1976). The chemical structure of prostaglandin X (prostacyclin). Prostaglandins.

[28] Coughlin D.J., Salomon R.G. (1977). Synthesis and thermal reactivity of some 2,3-dioxabicyclo[2.2.1]heptane models of prostaglandin endoperoxides. J. Am. Chem. Soc..

[29] Salomon R.G., Salomon M.F. (1977). 2,3-Dioxabicyclo[2.2.1]heptane-strained bicyclic peroxide nucleus of prostaglandin endoperoxides. J. Am. Chem. Soc..

[30] Zagorski M.G., Salomon R.G. (1980). Prostaglandin endoperoxides. 11. Mechanism of amine-catalyzed fragmentation of 2,3-dioxabicyclo[2.2.1]heptane. J. Am. Chem. Soc..

[31] Zagorski M.G., Salomon R.G. (1982). Prostaglandin endoperoxides. 12. Carboxylate catalysis and the effects of proton donors on the decomposition of 2,3-dioxabicyclo[2.2.1]heptane. J. Am. Chem. Soc..

[32] Iyer R.S., Miller D.B., Salomon R.G. (1990). Prostaglandin endoperoxides. 26. Decomposition of levuglandin-E2-dehydration and allylic rearrangement products. J. Org. Chem..

[33] Salomon R.G., Miller D.B., Raychaudhuri S.R., Avasthi K., Lal K., Levison B.S. (1984). Prostaglandin endoperoxides. 15. Asymmetric total synthesis of levuglandin E2. J. Am. Chem. Soc..

[34] Nugteren D.H., Vonkeman H., Vandorp D.A. (1967). Non-enzymic conversion of all-*cis* 8,11,14-eicosatrienoic acid into prostaglandin E1. Recueil des Travaux Chimiques des Pays-Bas.

[35] Morrow J.D., Minton T.A., Mukundan C.R., Campbell M.D., Zackert W.E., Daniel V.C., Badr K.F., Blair I.A., Roberts L.J. (1994). Free radical-induced generation of isoprostanes *in-vivo*—Evidence for the formation of D-ring and E-ring isoprostanes. J. Biol. Chem..

[36] Morrow J.D., Awad J.A., Wu A.P., Zackert W.E., Daniel V.C., Roberts L.J. (1996). Nonenzymatic free radical-catalyzed generation of thromboxane-like compounds (isothromboxanes) *in vivo*. J. Biol. Chem..

[37] Salomon R.G. (1985). Prostaglandin endoperoxide reaction-mechanisms and the discovery of levuglandins. Acc. Chem. Res..

[38] Hobbs H.H., Russell D.W., Brown M.S., Goldstein J.L. (1990). The LDL receptor locus in familial hypercholesterolemia: Mutational analysis of a membrane protein. Annu. Rev. Genet..

[39] Bernoud-Hubac N., Davies S.S., Boutaud O., Montine T.J., Roberts L.J. (2001). Formation of highly reactive gamma-ketoaldehydes (neuroketals) as products of the neuroprostane pathway. J. Biol. Chem..

[40] Zhang R.L., Brennan M.L., Shen Z.Z., MacPherson J.C., Schmitt D., Molenda C.E., Hazen S.L. (2002). Myeloperoxidase functions as a major enzymatic catalyst for initiation of lipid peroxidation at sites of inflammation. J. Biol. Chem..

[41] Klebanoff S.J., Clark R.A. (1978). The Neutrophil: Function and Clinical Disorders.

[42] Daugherty A., Dunn J.L., Rateri D.L., Heinecke J.W. (1994). Myeloperoxidase, a catalyst for lipoprotein oxidation, is expressed in human atherosclerotic lesions. J. Clin. Invest..

[43] Hazell L.J., Arnold L., Flowers D., Waeg G., Malle E., Stocker R. (1996). Presence of hypochlorite-modified proteins in human atherosclerotic lesions. J. Clin. Invest..

[44] Hazen S.L., Heinecke J.W. (1997). 3-Chlorotyrosine, a specific marker of myeloperoxidase-catalyzed oxidation, is markedly elevated in low density lipoprotein isolated from human atherosclerotic intima. J. Clin. Invest..

[45] Harrison J.E., Schultz J. (1976). Studies on the chlorinating activity of myeloperoxidase. J. Biol. Chem..

[46] Heinecke J.W., Li W., Daehnke H.L., Goldstein J.A. (1993). Dityrosine, a specific marker of oxidation, is synthesized by the myeloperoxidase-hydrogen peroxide system of human neutrophils and macrophages. J. Biol. Chem..

[47] Brennan M.L., Wu W., Fu X., Shen Z., Song W., Frost H., Vadseth C., Narine L., Lenkiewicz E., Borchers M.T. (2002). A tale of two controversies: defining both the role of peroxidases in nitrotyrosine formation *in vivo* using eosinophil peroxidase and myeloperoxidase-deficient mice, and the nature of peroxidase-generated reactive nitrogen species. J. Biol. Chem..

[48] van der Vliet A., Eiserich J.P., Halliwell B., Cross C.E. (1997). Formation of reactive nitrogen species during peroxidase-catalyzed oxidation of nitrite. A potential additional mechanism of nitric oxide-dependent toxicity. J. Biol. Chem..

[49] Carr A.C., McCall M.R., Frei B. (2000). Oxidation of LDL by myeloperoxidase and reactive nitrogen species: reaction pathways and antioxidant protection. Arterioscler. Thromb. Vasc. Biol..

[50] Podrez E.A., Abu-Soud H.M., Hazen S.L. (2000). Myeloperoxidase-generated oxidants and atherosclerosis. Free Radic. Biol. Med..

[51] Anderson G.J., Connor W.E., Corliss J.D. (1990). Docosahexaenoic acid is the preferred dietary N-3 fatty-acid for the development of the brain and retina. Pediatr. Res..

[52] Connor W.E., Neuringer M., Lin D.S. (1990). Dietary effects on brain fatty acid composition: The reversibility of n-3 fatty acid deficiency and turnover of docosahexaenoic acid in the brain, erythrocytes, and plasma of rhesus monkeys. J. Lipid Res..

[53] Connor W.E., Neuringer M., Reisbick S. (1992). Essential fatty-acids—The importance of N-3 fatty-acids in the retina and brain. Nutr. Rev..

[54] Anderson R.E., Rapp L.M., Wiegand R.D. (1984). Lipid-peroxidation and retinal degeneration. Curr. Eye Res..

[55] Halliwell B. (1992). Reactive oxygen species and the central-nervous-system. J. Neurochem..

[56] Simonian N.A., Coyle J.T. (1996). Oxidative stress in neurodegenerative diseases. Ann. Rev. Pharm. Toxicol..

[57] Knight J.A. (1997). Reactive oxygen species and the neurodegenerative disorders. Ann. Clin. Lab. Sci..

[58] Markesbery W.R. (1997). Oxidative stress hypothesis in Alzheimer’s disease. Free Radic. Biol. Med..

[59] Roberts L.J., Montine T.J., Markesbery W.R., Tapper A.R., Hardy P., Chemtob S., Dettbarn W.D., Morrow J.D. (1998). Formation of isoprostane-like compounds (neuroprostanes) *in vivo* from docosahexaenoic acid. J. Biol. Chem..

[60] Nourooz-Zadeh J., Liu E.H.C., Anggard E.E., Halliwell B. (1998). F-4-isoprostanes: A novel class of prostanoids formed during peroxidation of docosahexaenoic acid (DHA). Biochem. Biophys. Res. Commun..

[61] Salomon R.G., Miller D.B., Zagorski M.G., Coughlin D.J. (1984). Solvent induced fragmentation of prostaglandin endoperoxides. New aldehyde products from PGH2 and a novel intramolecular 1,2-hydride shift during endoperoxide fragmentation in aqueous solution. J. Am. Chem. Soc..

[62] Salomon R.G., Jirousek M.R., Ghosh S., Sharma R.B. (1987). Prostaglandin endoperoxides 21. Covalent binding of levuglandin E2 with proteins. Prostaglandins.

[63] Brame C.J., Salomon R.G., Morrow J.D., Roberts L.J. (1999). Identification of extremely reactive gamma-ketoaldehydes (isolevuglandins) as products of the isoprostane pathway and characterization of their lysyl protein adducts. J. Biol. Chem..

[64] Boutaud O., Brame C.J., Salomon R.G., Roberts L.J., Oates J.A. (1999). Characterization of the lysyl adducts formed from prostaglandin H2 via the levuglandin pathway. Biochemistry.

[65] Iyer R.S., Kobierski M.E., Salomon R.G. (1994). Generation of pyrroles in the reaction of levuglandin E2 with proteins. J. Org. Chem..

[66] Bernoud-Hubac N., Fay L.B., Armarnath V., Guichardant M., Bacot S., Davies S.S., Roberts L.J., Lagarde M. (2004). Covalent binding of isoketals to ethanolamine phospholipids. Free Radic. Biol. Med..

[67] Difranco E., Subbanagounder G., Kim S., Murthi K., Taneda S., Monnier V.M., Salomon R.G. (1995). Formation and stability of pyrrole adducts in the reaction of levuglandin E(2) with proteins. Chem. Res. Toxicol..

[68] Kobierski M.E., Kim S.C., Murthi K.K., Iyer R.S., Salomon R.G. (1994). Synthesis of a pyrazole isostere of pyrroles formed by the reaction of the epsilon-amino groups of protein lysyl residues with levuglandin E(2). J. Org. Chem..

[69] Davies S.S., Amarnath V., Roberts L.J. (2004). Isoketals: Highly reactive gamma-ketoaldehydes formed from the H2-isoprostane pathway. Chem. Phys. Lipids.

[70] Yamashita M., Fenn J.B. (1984). Electrospray ion-source—Another variation on the free-jet theme. J. Phys. Chem..

[71] Tanaka K., Waki H., Ido Y., Akita S., Yoshida Y., Yoshida T., Matsuo T. (1988). Protein and polymer analyses up to *m*/*z* 100 000 by laser ionization time-of-flight mass spectrometry. Rapid Commun. Mass Spectrom.

[72] Karas M., Hillenkamp F. (1988). Laser desorption ionization of proteins with molecular masses exceeding 10000 Daltons. Anal. Chem..

[73] Domingues M.R.M., Reis A., Domingues P. (2008). Mass spectrometry analysis of oxidized phospholipids. Chem. Phys. Lipids.

[74] Li W. (2009). Oxidative modification of ethanolamine phospholipids by isolevuglandins: Detection by LC-MS/MS *in vitro* and *in vivo*. Ph.D. Thesis.

[75] Brame C.J., Boutaud O., Davies S.S., Yang T., Oates J.A., Roden D., Roberts L.J. (2004). Modification of proteins by isoketal-containing oxidized phospholipids. J. Biol. Chem..

[76] Boutaud O., Li J., Zagol I., Shipp E.A., Davies S.S., Roberts L.J., Oates J.A. (2003). Levuglandinyl adducts of proteins are formed via a prostaglandin H2 synthase-dependent pathway after platelet activation. J. Biol. Chem..

[77] Li W., Laird J.M., Lu L., Roychowdhury S., Nagy L.E., Zhou R., Crabb J.W., Salomon R.G. (2009). Isolevuglandins covalently modify phosphatidylethanolamines *in vivo*: Detection and quantitative analysis of hydroxylactam adducts. Free Radic. Biol. Med..

[78] Roychowdhury S., McMullen M.R., Pritchard M.T., Li W., Salomon R.G., Nagy L.E. (2009). Formation of gamma-ketoaldehyde-protein adducts during ethanol-induced liver injury in mice. Free Radic. Biol. Med..

